# Spatiotemporal heterogeneity of the association between socioeconomic development and birth rate: a geographically and temporally weighted regression modeling study in China

**DOI:** 10.3389/fpubh.2025.1587358

**Published:** 2025-05-21

**Authors:** Yu Yang, Rongxin He, Liming Li

**Affiliations:** ^1^School of Humanities and Social Sciences, Xi’an Jiaotong University, Xi’an, China; ^2^School of Health Management, Southern Medical University, Guangzhou, China

**Keywords:** spatiotemporal heterogeneity, socioeconomic development, birth rate, GTWR, population policy

## Abstract

**Background:**

The birth rate is an important indicator of the health of the population. However, persistently low birth rate has become a pressing demographic challenge for many countries, including China. This has significant implications for sustainable population planning.

**Methods:**

This study applied hot spot analysis and the spatiotemporal geographically weighted regression (GTWR) modeling, used panel data of 286 cities in China from 2012 to 2021 to explore the spatiotemporal heterogeneity of the relationship between the socioeconomic development and birth rate.

**Results:**

The research has found that 2017 was an important turning point in China’s demographic transition. The hot spot analysis reveals that the birth rate hot spots are characterized by a multipolar kernel distribution, shifting from spatial diffusion to convergence, with the cold spots mainly located in the northeast. And the GTWR modeling found that the relationship between socioeconomic development and birth rate varies and change dynamically over space and time. Key findings include: (1) the negative impact of GDP per capita on birth rates has intensified; (2) housing prices exhibit both wealth and crowding-out effects on birth rates, and there are obvious regional differences between the north and the south; (3) fiscal education expenditure on birth rates has the most pronounced income effect in the eastern region.

**Conclusion:**

This study adopts spatiotemporal perspective to reveal the spatiotemporal heterogeneity of the association between socioeconomic development and birth rate. It provides new evidence on the influence of macro factors on fertility in China. And emphasizes the importance of incorporating regional variations into population policy design.

## 1 Introduction

The demographic transition refers to the shift from a traditional to a modernized society, marked by declines in both birth and death rates (1). In recent years, more than 40 countries or regions have experienced negative population growth (2). The World Population Prospects 2022 indicates that the populations of 61 countries or regions are projected to decline by 1% or more between 2022 and 2050. (3). Low birth rates contribute to several societal issues, including a shrinking working-age population, demographic imbalances, higher retirement burdens, slower GDP growth, and an aging population, all of which hinder development (4, 5). As economies develop, families tend to reduce their demand for children, and early declines in birth rates were primarily observed in developed countries (6, 7). However, this trend is now extending to developing countries like China, India, and Egypt (8). In China, despite government policies encouraging higher birth rates, the natural population growth rate dropped to-1.48‰ by the end of 2023 (9) posing risks to the country’s future development.

Extensive research has been conducted on low birth rates in developed countries. It is widely argued that changes in birth and death rates reflect the demographic transition (10). The basic viewpoint of demographic transition theory holds that the population development are intrinsically linked to socioeconomic development rather than occurring in isolation (11). Empirical studies have identified multiple influencing factors, including economic instability (12), public education, social security (13) house affordability (11, 14). Studies have shown that that government efforts to create a favorable social environment for childbearing can effectively boost birth rates (15). For instance, empirical studies has demonstrated that the Clean Air Act Amendments of 1970 in the United States significantly reduced neonatal mortality rates. (16). However, the decline in China’s fertility rate was driven not only by economic growth but also by stringent population control policies. Consequently, China’s demographic transition has been more rapid than that of other developed countries (17). In terms of economic development, studies demonstrate that fertility in China is influenced by societal changes (18), education and urbanization (19), income, improvements in social security (20), housing affordability and wealth (21, 22) and public health services (23). However, China has experienced fertility policy changed and regional development imbalances in the last decade. Uneven socioeconomic development levels, coupled with the variations of implementation and enforcement of family planning policies, could lead to fertility variations across space (24). Several studies have identified significant spatial variations in socioeconomic factors and birth rates (25, 26) in developed countries, offering new perspectives to address this issue. Although a limited number of studies have highlighted the spatial and temporal variations in birth rates across China (5, 24), there are limitations of under-capturing macro factors and time effects. Furthermore, provincial-level analyses often mask significant intra-provincial differences among cities. The fundamental units of public policy implementation, cities possess distinct economic and social structures that profoundly influence birth rates and warrant closer attention.

To fill this gap, it is essential to consider both a spatiotemporal perspective and captures the impact of socioeconomic development on the birth rate, and respects China’s national conditions by conducting the study on a city-by-city basis. Thereby, this study adopts a spatiotemporal perspective and employs a 10-year city-level panel dataset to explore the temporal and spatial heterogeneity of the relationship between socioeconomic development and birth rates. Thereby distinguishing it from most previous research.

## 2 Methods

### 2.1 Data

This study includes data from 286 cities in China. After the excluding missing values, the final sample size (N = 2,648) comprises diverse sources, including the China Urban Statistical Yearbook (2013–2022), the China Regional Economic Statistics Yearbook (2013–2014), and the Statistical Bulletin of National Economic and Social Development (2012–2021). The variables considered include the Birth Rate, Per capita GDP (PGDP), Urbanization Rate (UR), Fiscal Science and Technology Expenditure (FSTE),Urban–Rural Income Gap (URIG), Air Quality Index (AQI), House Prices (HP), Hospital beds per 1,000 people (HBPT), Fiscal Education Expenditure (FEE), and the number of public library books per 100 people (PBPH). Notably, ethnic minority autonomous regions are excluded due to disparities in fertility policies compared to the rest of China. For ethnic groups exceeding ten million, fertility restrictions remain relatively strict yet more lenient than Han standards. Those below 10 million are permitted two or three children depending on residential areas and specific conditions, while groups with extremely small populations face no restrictions. These inherent policy disparities prevent unified evaluation frameworks across regions. Moreover, there is a severe lack of data on birth rates in ethnic minority regions. Therefore, we have not included these regions in our analysis. However, studies have shown that as of 2020, the number of live-born children for all ethnic minorities is below the replacement level (27). This indicates that ethnic minorities have also experienced low fertility, which is consistent with other regions in China.

### 2.2 Variables

Human behavior is intricately shaped by the social environment. Fertility, whether viewed as an individual or familial behavior, is influenced by various social factors. China’s negative population growth is driven by persistently low birth rates, attributed to significant changes in the macro-level fertility environment (28). Assuming that the macro-level social environment significantly influences fertility behavior, we construct socioeconomic development across five domains: (1) economic level; (2) housing level; (3) environmental level; (4) medical level; and (5) education level.

The economic level include GDP per capita (PGDP), urbanization rate (UR), urban–rural income gap (URIG),and fiscal science and technology expenditure (FSTE); The housing level include house price (HP); The environmental level include air quality index (AQI); The medical level include hospital beds per 1,000 people (HBPT); and The educational level include fiscal education expenditure (FEE), and public library books per 100 people (PBPH). For a detailed summary in Table 1.

**Table 1:** Variable summary (*n* = 2,648).

Socioeconomic development	Variable		Mean value	Standard deviation
	Dependent variable			
	Birth Rate	Birth rate = (Births per year / Average annual population)*1,000‰	10.9257	3.42152
	Independent variable			
Economic level	PGDP	Ln of GDP per capita (10, 000 yuan)	1.5725	0.5620
	UR	Urban population/Total population (%)	0.5635	0.1466
	URIG	The per capita urban disposable income/ The per capita rural disposable income (%)	2.3126	0.4491
	FSTE	Fiscal science and technology expenditure as a share of public expenditure (%)	1.7203	1.7666
Environmental level	AQI	Ln of air quality index	3.6856	0.3862
Housing level	HP	Ln of house price (yuan/m^2^)	8.5894	0.5105
Educational level	FEE	Fiscal education expenditure as a share of public expenditure (%)	17.5582	3.9247
	PBPH	Ln of the number of public library books per 100 people	3.8781	0.7956
Medical level	HBPT	Ln of hospital beds per 1,000 people	3.8223	0.3044

#### 2.2.1 Birth rate

This study uses the birth rate as the dependent variable. The birth rate is defined as the average number of births per 1,000 people in a given area over a certain period (usually 1 year). It serves as a fundamental indicator of fertility level and natural population changes in a country or region.

**Figure 1:**
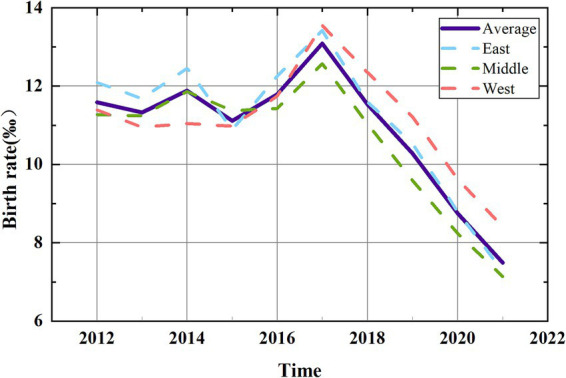
Birth rate in China (2012–2021).

As shown in Figure 1, China’s birth rate transitioned from post-2012 fluctuations to a steady post-2017 decline, signaling a critical demographic shift. Between May 30, 2014, and January 1, 2016, the One-Child Policy was relaxed to allow couples to have two children if either spouse was an only child (OTCP). The Universal Two-Child Policy (UTCP) allowed every couple to have two children since January 1, 2016 (29). Due to the time required for pregnancy and childbirth, the effects of such policies exhibit a lag. Figure 1 reveals that births remained low until 2016, indicating limited impact from OTCP. Following UTCP, the birth rate peaked in 2017 but subsequently declined annually, suggesting that UTCP had a strong short-term effect that diminished over time. These policy adjustments highlight that while fertility policies can boost birth rates temporarily, their long-term sustainability is limited. Thus, other structural factors must be considered. Additionally, regional disparities show sharper declines in the eastern and central regions compared to the west, necessitating region-specific analyses. In this study, we categorize birth rate trends into two phases: the Fluctuation Phase (2012–2016) and the Downward Phase (2017–2021). This division enables a more detailed analysis of how socioeconomic variables impacts birth rates over time.

#### 2.2.2 Economic level

The economic base serves as a crucial measure of a nation’s economic strength. It directly influences the country’s international status, the quality of life of its people, and societal stability and development. Research has demonstrated a significant negative correlation between economic development and fertility (30). Specifically, countries with higher PGDP tend to experience lower birth rates (31). As economic conditions improve, families tend to prioritize the quality of children over quantity, leading to reduced birth rates (7). Additionally, China’s inherent urban–rural dual structure, the widening gap between urban and rural areas and the constant upgrading urbanization rate all shape fertility decisions, further contributing to lower birth rates (32, 33). Thus, we hypothesize that PGDP, URIG, and UR are negatively correlated with the birth rate. Besides, FSTE is a key method for government support of regional innovation and contributes to the coordinated development of regional economies (34). Therefore, we hypothesize that FSTE has a positive impact on the birth rate.

#### 2.2.3 Housing level

The affordability of HP significantly influences individuals’ reproductive choices. An optimal housing market is characterized by households having access to suitable housing at a reasonable cost (35). Studies indicate a strong correlation between childbirth and residential mobility. Couples with a strong desire to have children are more inclined to relocate to areas with affordable housing options (36). Families with children may prefer larger homes, secure leases, and favorable locations compared to households without children (37). Furthermore, housing size is the housing factor most significantly linked to the timing of first births. High housing costs contribute to postponing a couple’s initial childbirth by approximately 3–4 years (38). In China, rising house prices have adversely impacted childbirth. Some studies underscore a negative childbearing response to the surge in house values driven by the recent housing boom in China (22). Therefore, we hypothesizes that the impact of HP on birth rates could have negative effects.

#### 2.2.4 Environmental level

The ecological environment is closely linked to people’s physical health. Among the various factors of environmental pollution, air pollution is particularly significant due to humanity’s continuous and vital need for oxygen (39). The AQI is an important indicator of air quality. Studies have shown that air pollution is a major contributor to human health issues, increased infertility, and declining population growth rates (40, 41). Evidence indicates that exposure to poor air quality during pregnancy raises the risk of adverse birth outcomes (42). Long-term exposure to polluted air also increases the risk of infertility and various cancer (41). Numerous studies have found that higher levels of air pollution are associated with decrease birth rates (43, 44). Therefore, we hypothesize that there is a negative correlation between AQI and birth rates.

#### 2.2.5 Medical level

With economic development, improvement of healthcare have significantly enhanced people’s health. According to data from the World Health Organization (WHO), the number of newborn deaths worldwide has decreased by 44% since 2000. Access to and availability of quality health care continues to be a matter of life or death for mothers and newborns globally (45). Adequate healthcare resources can enhance the accessibility of healthcare resources for people. Studies show that the majority of women in the United States consider prenatal and postnatal medical check-ups essential to routine maternal and infant care (46). In China, maternity check-ups have become a new ethic of health responsibilities for pregnant women (47). Over the past few decades, China’s ongoing efforts in the health system, particularly in the field of reproductive, maternal, newborn, child, and adolescent health (RMNCAH), have achieved significant milestones (48). These initiatives have ensured that the health needs of women and children are met. Research indicates that improved medical security and increased infant survival rates have reduced the incentive for families to have more children as a buffer against mortality rates (49). Therefore, we hypothesize HBPT will reduce the number of children people have, further lowering the birth rates.

#### 2.2.6 Education level

In household economics, there are two relationships between FEE and birth rate. On one hand, increased FEE will reduce burden on families, encouraging them to have more children through an “income effect.” On the other hand, FEE can lower the cost of human capital investment for households, prompting them to substitute quantity of children with quality through a “substitution effect.” (50). In East Asian societies, there is a high emphasis on children’s education, making it common for families to increase their investment in this area (51). Enhanced FEE can reduce parental anxiety regarding their children’s education (52) and improve their fertility intention (53). If households interpret public spending on education as a fertility subsidy within their decision-making model, increase spending can effectively promotes social childbirth (54).Besides, public libraries are among key sites for the acquisition of cultural and digital resources (55).This is also a way for cities to provide socialized education, which is why we include PBPH within the scope of education level. Therefore, we hypothesize that the impact of FEE and PBPH on birth rates are positive.

### 2.3 Spatiotemporal regression modeling

Considering the dynamic nature of the birth rate and the significant geographical disparities in China, conventional regression models can only capture average effects, neglecting spatial and temporal heterogeneity. The Geographically and Temporally Weighted Regression (GTWR), an extended GWR model, addresses this limitation. It enables the incorporation of time effects into geographically weighted models, effectively addressing issues of temporal and spatial non-stationarity in the data simultaneously (56, 57). This methodology, extensively applied in real estate (56, 57), environmental pollution (58, 59), urban vitality (60), and other studies. Some studies have consistently found that the GTWR model produced a better model fit than traditional regression models. A typical GTWR model can be written as follows:


$$Y_{i}=\beta_{0}(u_{i},v_{i},t_{i})+\sum_{k}\beta_{k}(u_{i},v_{i},t_{i})X_{\mathrm{ik}}+\varepsilon_{i}$$

Let Y_i_ denote the dependent variable, the birth rate of city i; (u_i_, v_i_, t_i_) denotes the spatial location (u_i_, v_i_ as coordinates) of census tract i at time t_i_; β0 (u_i_, v_i_, t_i_) is the intercept value; βk (u_i_, v_i_, t_i_) represents a vector of parameter value for the independent variable k at the census tract I, and X_ik_ is the respective independent variable; and ε_i_ denotes an error term for census tract i. What is distinct about the GTWR model is that it allows the parameters β_k_ (u_i_, v_i_, t_i_) to vary across the model to measure both the spatial and temporal variations in a spatiotemporal dataset. To calibrate this model, a spacetime weight matrix W (u_i_, v_i_, t_i_), a diagonal matrix with elements representing the spatial and temporal weights of each census tract i, is required. The optimal spatiotemporal weight matrix is determined through a cross-validation (CV) approach, seeking the best goodness of fit. This calibration process employs the local weighted least squares approach in conjunction with the GTWR model. The articles Huang et.al (56) and Fotheringham et al. (57) provide detailed discussions on GTWR model calibration.

Moran’s I was computed for the dataset, revealing a value of 0.519 (*p* < 0.000), signifying robust spatial autocorrelation and indicating the necessity of employing a spatial regression approach. A spatial non-stationarity test was performed by comparing the interquartile range from the GTWR with twice the standard errors from the Ordinary Least Squares (OLS) model (Supplementary Table S1). The results indicated noticeable extra local variations in all variables, rendering GTWR more suitable for exploring spatiotemporal heterogeneity (57). Additionally, the GTWR model exhibited a higher adjusted R-squared of 0.64, surpassing the OLS model values of 0.25, respectively (Supplementary Table S2). This suggests that the GTWR model significantly enhances the overall performance of the model in capturing spatial and temporal variations within the research sample.

## 3 Results

### 3.1 Spatial characteristics of birth rate across the study period

**Figure 2:**
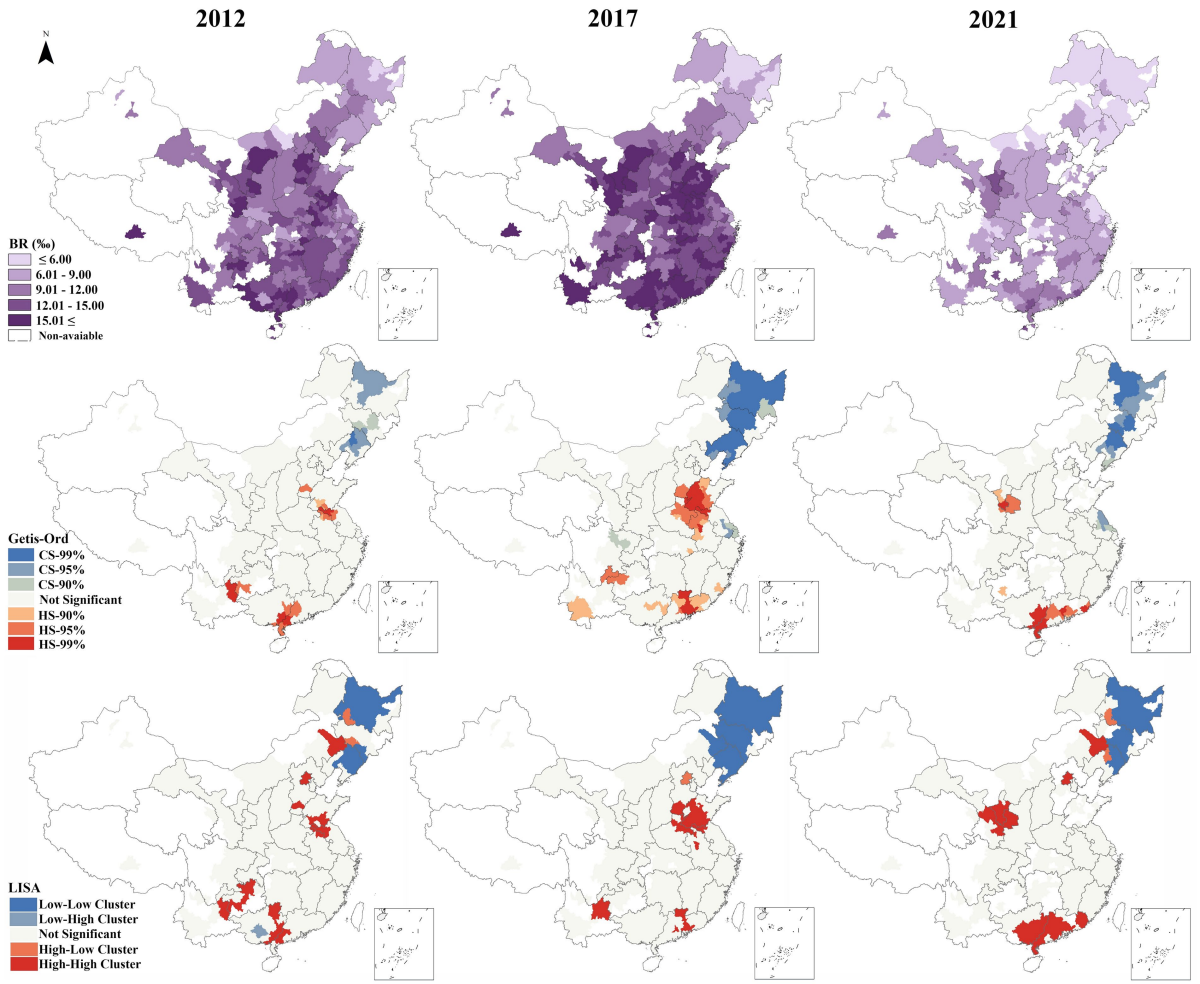
Spatial pattern of birth rate in all time.

The spatial distribution of China’s birth rate from 2012 to 2021 is depicted in Figure 2, revealing distinct spatial patterns characterized by an initial rise followed by a decline. The Northeast region exhibits the lowest birth rate compared to others regions. In contrast, the birth rates in the Northwest, North, Southwest, Central and South, and East China display varying degrees of increase and decrease. Temporally, with 2017 as the demarcation point, China’s birth rate demonstrates a growth trend from 2012 to 2016, followed by a decline from 2017 to 2021. Notably, in 2021, all regions of the country exhibit lower birth rates than in 2012.

This study employs the ArcGIS spatial hotspot analysis tool to identify the distribution of hot spots and cold spots in China’s birth rate. Hot spots represent clusters of high birth rate values, while cold spots indicate clusters of low values. As shown in Figure 2, hot spots are characterized by a multipolar kernel distribution, shifting from spatial diffusion to convergence, with the cold spots mainly located in the northeast. The study further confirmed the findings using Local Indicators of Spatial Association (LISA). Results demonstrated that high-high (HH) and low-low (LL) clusters closely aligned with hotspot and coldspot distributions, reinforcing the study’s conclusions.

We calculated the Moran’s I index for each year (Supplementary Table S3). The results showed that spatial autocorrelation gradually strengthened over time. Given that the response to fertility policies requires time, we designated the year 2017 as the first year after two-child policy. We followed the method of Omar El Deeb (61) and calculated the Moran’s I index before and after the policy implementation. The results show that the Moran’s I index before the policy (2012–2016) was 0.4589 (*p* < 0.000), while after the policy (2017–2021) it increased to 0.6063 (p < 0.000), representing 32.1% rise. This demonstrates heightened spatial inequality in birth rates following the two-child policy implementation. The LISA map (Figure 2) illustrates that in 2012 (pre-policy), a few regions exhibited HH clustering, while Heilongjiang Province displayed LL clustering. Scattered outliers “high-low” (HL) were minimal, suggesting a spatially continuous birth rate distribution with no significant local anomalies. During this period, China maintained the “one-child policy,” which tightly regulated fertility levels, with regional variations primarily driven by socioeconomic and cultural factors. In 2017 (1 year post-implementation of two-child policy), the expansion of both HH and LL clusters highlighted the policy’s spatially heterogeneous initial impact. Regions with higher fertility intentions exhibited rapid responsiveness to the policy, while persistent low fertility persisted in northeastern provinces, underscoring uneven policy effectiveness. By 2021 (5 years post-implementation of two-child policy), HH clusters expanded westward, with eastern HH clusters diminishing. While LL clusters in northeastern provinces contracted but persisted significantly. This highlights the structural nature of low fertility and its resistance to short-term reversal.

Northeast is the region with the number of government or state-owned institutions in China. This region has enforced the one-child policy most rigorously (62). So, the concept of having fewer children has been widely accepted and implemented. Over the past decade, the economy in the Northeast has not fared as well, leading to a significant outflow of young people (63). Consequently, this has resulted in a decline in the population of individuals of childbearing age. These may be the reasons why birth rate cold spots are concentrated in the northeast.

The emergence of fertility hotspots indicates that UTCP has significantly boosted the birth rate in certain regions. The fertility restriction policies had suppressed the reproductive desires of a portion of the population, leading to the accumulation of a strong willingness to have children. Once the policies were relaxed, these pent-up desires were released in a concentrated manner (64). However, as the demand for a second child is gradually met, the fertility hotspots are diminishing in number and scope, signifying that the short-term effects of the fertility policy are waning and population growth is gradually returning to a normal pattern.

### 3.2 Geographically and temporally weighted regression modeling

#### 3.2.1 Overall model

The GTWR model results, as shown in Table 2, reveals distinct correlations between birth rate and socioeconomic development. On average, in the realm of economic security, PGDP exhibits a negative correlation with the birth rate, while the UR, URIG, and FSTE demonstrate positive correlations with the birth rate. Regarding housing security, an increase in HP corresponds to a rise in the birth rate. In terms of environmental protection, there is a positive correlation between AQI and the birth rate. In the domain of medical security, the availability of HBPT shows a negative correlation with the birth rate, indicating that abundance of HBPT is associated with a lower birth rate. Within educational security, FEE positively impacts the birth rate. Conversely, PBPH exhibits a negative correlation with the birth rate.

**Table 2:** GTWR model summary.

Variable	AVG	MIN	LQ	MED	UQ	MAX
PGDP	−0.78141	−3.82378	−1.80789	−0.78777	0.314961	9.237364
UR	2.765735	−15.5702	−0.20312	2.658257	6.019264	11.09061
URIG	1.225326	−0.82544	0.68043	1.193693	1.753461	8.459487
FSTE	0.145841	−0.63771	−0.02387	0.152916	0.292711	4.413837
HP	0.39278	−7.01649	−0.34651	0.154343	1.259822	4.24034
AQI	0.798855	−4.84081	−0.45069	0.878487	1.931379	4.785084
HBPT	−1.63736	−6.93042	−2.61386	−1.435	−0.76472	5.813441
FEE	0.092567	−1.09561	0.008628	0.102924	0.18716	0.355683
PBPH	−0.09587	−6.00907	−0.37928	−0.0751	0.166508	1.883449
Adjusted R^2^	0.637209					

Table 3 presents the average coefficients illustrating the impact of each variable on the birth rate during the Fluctuation Phase (2012–2016) and Downward Phase (2017–2021), further supporting our analysis. The comparison between the two phases reveals that economic development (PGDP) and rising HP may have initially promoted the birth rate, but their suppressive effects became stronger in the later phase. UR and the urban–rural URIG had a positive impact on the birth rate, especially during the declining phase. Increased FSTE and FEE positively influenced the birth rate in the later phase. Improved air quality (AQI) also had a positive effect on the birth rate in the later phase, while the increase HBPT may have suppressed it. The growth in PBPH positively affected the birth rate in the later phase. Overall, the results in Table 3 indicate that the impact of different factors on the birth rate varied significantly across phases, particularly during 2017–2021. And UR, FSTE, FEE, AQI, and PBPH had more pronounced positive effects on the birth rate.

**Table 3:** Average coefficients in different periods.

Variable	Fluctuation phase (2012–2016)	Downward phase (2017–2021)	Average coefficient change
PGDP	−0.50494	−1.1214	−0.61646
UR	2.120463	3.420526	1.300062
URIG	0.618393	1.765849	1.147456
FSTE	0.006086	0.250689	0.244603
HP	0.621529	0.25997	−0.36156
AQI	0.164764	1.565107	1.400343
HBPT	−1.34859	−1.92803	−0.57944
FEE	0.020142	0.168103	0.147961
PBPH	−0.22116	0.012384	0.23354

#### 3.2.2 Temporal dimension

**Figure 3:**
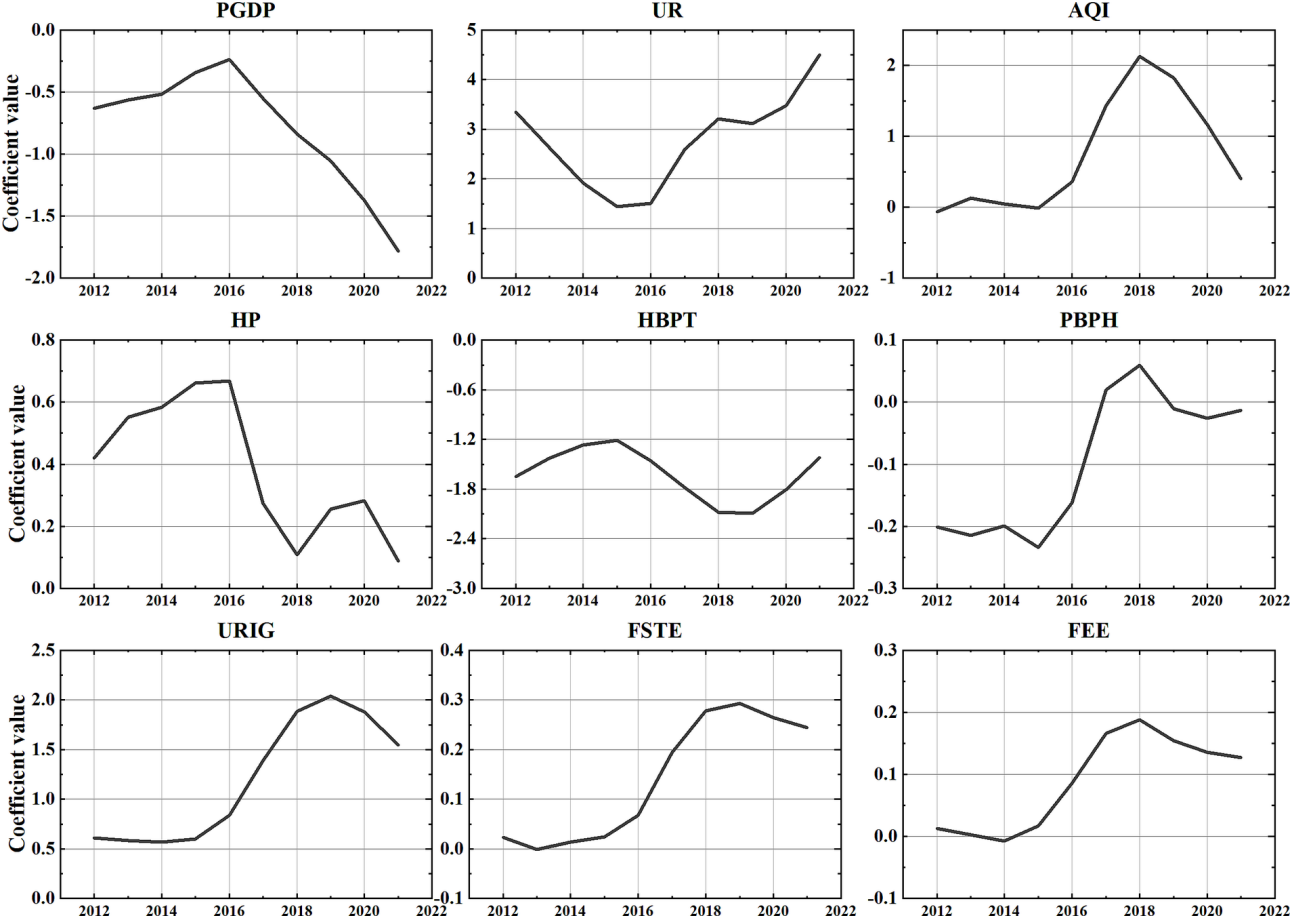
Temporal trends in the impact of each variable on the birth rate from 2012 to 2021.

Leveraging the strengths of the GTWR model, we obtained the coefficient time series for various stages of the study and created Figure 3. Which provides a detailed depiction of the temporal trends in the impact of each variable on the birth rate from 2012 to 2021. The adverse influence of PGDP on the birth rate shows a notable increase each year post-2016. The positive impact of the UR experiences fluctuations but maintains an overall ascending trend. Around 2018, the positive impact of AQI undergoes significant changes. The positive effect of HP on the birth rate fluctuates and diminishes, with a more pronounced weakening during the period of 2016–2018. The HBPT follows a wavering pattern. Notably, the effect of PBPH on the birth rate shifts from negative to positive after 2017. Furthermore, URIG, FEE, and FSTE exhibit similar trends, albeit with variations in the degree of positive enhancement.

As shown in Table 3 and Figure 3, the research results are generally consistent with the hypotheses derived from the literature, although there are also some exceptions. Specifically, our hypotheses regarding the impact of PGDP, HBPT, FSTE, and FEE on the birth rates have all been confirmed.

#### 3.2.3 Spatial dimension

**Figure 4:**
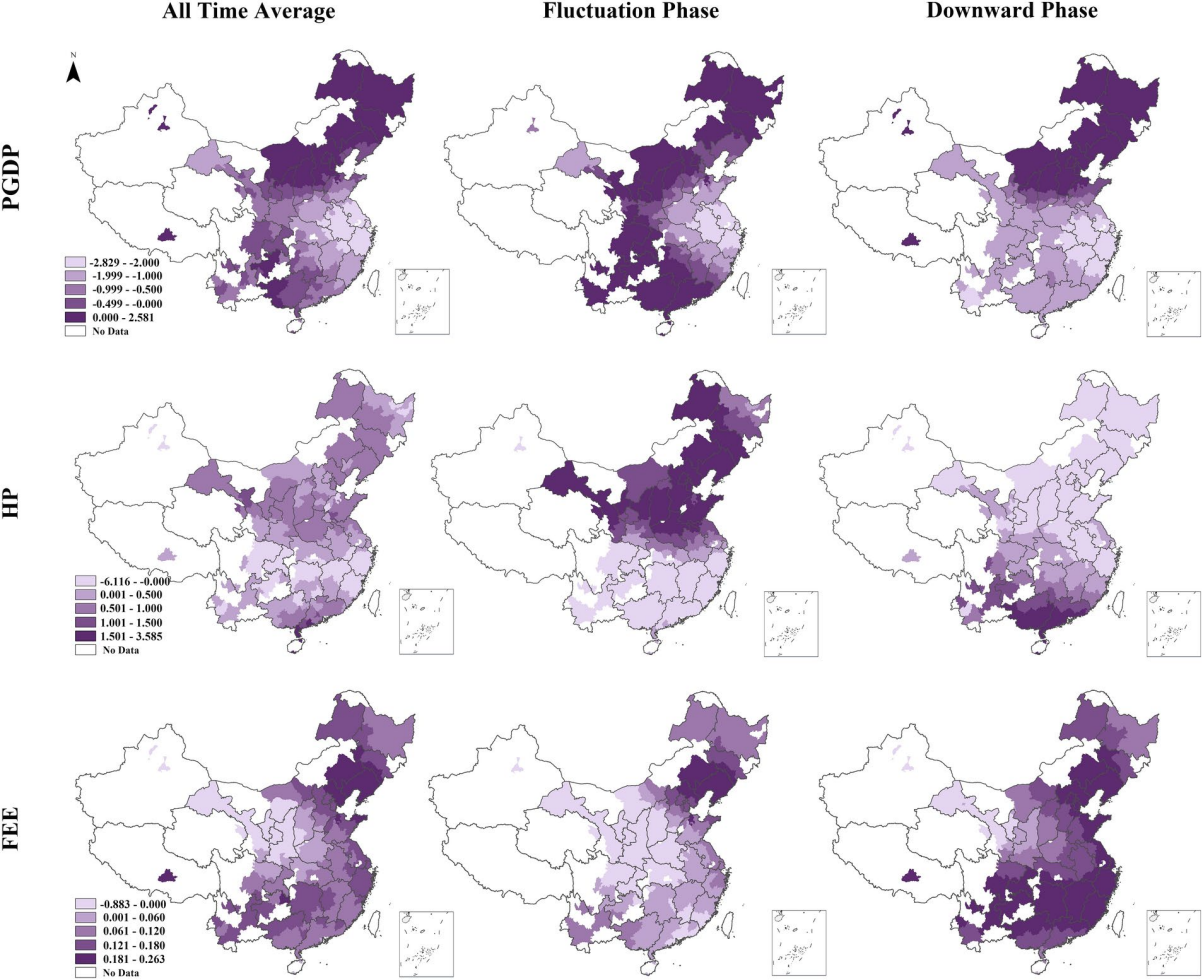
Spatial pattern of the average coefficients for three variables in all time, fluctuation phase, and downward phase.

Utilizing the spatial mappability inherent in the GTWR model, this study aims to explore the spatial relationships of each factor with the birth rate. Consideration space limitations, the paper chooses to conduct a focused analysis of representative factors from economic security, housing security, and education security. Specifically, PGDP, HP, and FEE are selected for detailed examination, as illustrated in Figure 4.

The average impact of PGDP on the birth rate was consistently significant nationwide throughout the study period. During the Fluctuation Phase, PGDP negatively affected birth rates only in parts of eastern (Shandong, Jiangsu, Shanghai, Anhui, Zhejiang) and central China (Henan, Hubei). In the Downward Phase, this negative impact expanded to include Central, Northwest, South, and Southwest China, with increasing intensity. This indicates that the negative impact of economic development on the population birth rate has gradually intensified over time and across space. The spatial distribution of HP’s impact on birth rates showed distinct patterns, with negative effects concentrated in regions like Zhejiang, Fujian, northern Jiangxi, northern Hunan, Chongqing, northern Guizhou, and eastern Sichuan. During the Fluctuation Phase, HP’s negative impact was primarily in southern regions, but it shifted northward in the Downward Phase, with its wealth effect weakening over time. This shows that the impact of HP on the population birth rate has significant regional differences. Moreover, as time progresses, the crowding-out effect gradually increases, while the wealth effect still persists. FEE’s correlation with birth rates displayed regional disparities, generally positive except in the northwest. In the Fluctuation Phase, northeastern and eastern regions showed positive effects, while in the Downward Phase, the positive effect became nationwide, excluding Gansu and Ningxia. This indicates that the impact of FEE on the birth rate is gradually manifesting its income effect.

## 4 Discussion

In temporal dimension section, we have demonstrated the trend of each variable’s impact on the birth rate over time. The average impact of PGDP is stable negative correlation with birth rate. This conclusion is consistent with the findings of many scholars (65, 66). The impact of HBPT on the birth rate, while showing corresponding fluctuations, is generally negatively correlated. This suggests that sufficient healthcare resources improve access to healthcare services for mothers and children, further reducing compensatory fertility (49). Increased investment in FSTE is conducive to coordinated regional economic development and provides a healthy socioeconomic environment for family reproduction. The average impact of FEE on birth rates is positive, which is consistent with the initial hypothesis. This suggests that increased FEE reduce the educational burden on families and promotes population birth (54, 67). Additionally, from the temporal dimension, this study also found that FEE exhibits an S-shaped fluctuation with the birth rate, aligning with previous research findings (68).

The impact of certain variables on birth rate has been unexpected. URIG is positively correlated with the birth rate. The possible reason is that URIG reflects the equilibrium of urban development. In China, cities with large urban–rural income gaps have relatively backward socio-economic development. According to international experience, regions with backward economic development are often areas with higher birth rates. Low-quality urbanization, characterized by urban population concentration without corresponding high-skilled job opportunities, has also been linked to higher fertility (69). AQI shows a positive correlation with birth rates, potentially influenced by regional and population differences. High AQI levels are typically associated with highly industrialized and economically vibrant regions (70). These areas attract a substantial influx of young labor migrants (71), increasing the proportion of the population of childbearing age and thereby elevating birth rates. PBPH negatively correlates with the birth rate. A possible reason is that areas with large library collections are often in big cities, where high childcare and living costs reduce people’s fertility desire. Conversely, this study also found a positive correlation between HP and birth rates. Some studies have shown that short-term surges in house price lead to a net increase in childbearing among those with housing (72). The rise in housing prices means an increase in family assets, which is conducive to promoting family fertility decisions (14), Similar conclusions have also been drawn in studies in China, which show that for every 1% increase in housing wealth, the fertility rate is likely to rise by 0.34% (22).

In Spatial dimension section, firstly, in terms of economic security, the relationship between PGDP and the birth rate generally exhibits a coexistence of negative correlation across the spatial dimension. This suggests that as the economy grows, the birth rate decreases. This finding corroborates the established negative relationship between economic growth and fertility (30). The two phases indicates that the rise PGDP is beneficial the increase in the birth rate in Northeast and North China. In southern regions, the relationship is more complex, showing a distinct spatial pattern. It has been shown that spatial fertility variations are contextual to the development stage of a given region (73). Additionally, China’s regional economic imbalances, with slower growth in the north and faster growth in the south (74). This created divergent development patterns, further influencing regional birth rates.

Secondly, regarding housing security, this study reveals that HP impact birth rates exhibits through both wealth and crowd-out effect in spatial terms. This finding is consistent with existing studies (75). Spatially, these effects exhibit distinct geographic patterns. From 2017 to 2021, a noticeable wealth effect emerged in the South, while a crowding-out effect in the North. The north–south disparity may be attributed to the mobility patterns of China’s population. Southern regions, historically exhibiting economic prowess, serve as a magnet for population influx (76). The migration of young laborers to the south not only fuels its economic growth but also influences birth decisions. Research indicates that families possessing property in migration destinations exhibit a greater desire to have second child, even amidst rising house price (75). Consequently, the South experiences rising housing prices alongside a large childbearing-age population, reflecting the wealth effect. In contrast, the North faces economic stagnation, youth outflows, an aging population, and rising housing costs, leading to a crowding-out effect on childbirth.

Finally, in the education security, the spatial impact of FEE on the birth rate reveal both substitution effect and income effect. As shown in Figure 4, the comparison of the two phases across the spatial dimension indicates that the positive effect of FEE on birth rates is gradually spreading and increasing. It began in the rapidly growing eastern part of the country and gradually spread from east to west over time. This may be attributed to the eastern region’s stronger economic foundation and larger fiscal budget, enabling higher FEE, which significant stimulating effect on the birth rate. The results also suggest that while FEE’s positive impact on birth rates may not be immediate, increasing FEE contributes to higher fertility in the long term.

## 5 Conclusion

This study reveals a significant correlation between socioeconomic development and birth rates. The hot spot analysis reveals that the birth rate hot spots are characterized by a multipolar kernel distribution, shifting from spatial diffusion to convergence, with the cold spots mainly located in the northeast. And through the GTWR model, this research also identifies spatiotemporal heterogeneity in the impact of socioeconomic development factors on birth rates. Specifically, as the economy develops, birth rate tend to be lower in cities with strong economies; The relationship between rising house price and birth rates shows both “wealth effect” and “crowding out effect” in the space; Increasing FEE promote birth rate in most areas of China. These research findings indicate that the impact of socioeconomic development on birth rates are characterized by multidimensionality, regional heterogeneity, and dynamic evolution. Therefore, when formulating and implementing population policies, it is necessary to consider regional development differences, the spatiotemporal heterogeneity of policy effects, and the interactions among different dimensions of socioeconomic development.

Based spatiotemporal perspective, this study provides new evidence on China’s birth rate trends, emphasizing the heterogeneity and clustering of influencing factors. To address declining birth rates, we recommend region-specific fertility policies. Key recommendations include: firstly, to focus on the birth rate cold spot. For instance, in the Northeast, where economic growth is slower and birth rates are significantly lower, the government should prioritize revitalizing the local economy to attract young people back and enhance their confidence in fertility. Secondly, When formulating fertility-promoting policies, local policymakers should consider the specific characteristics of local fertility dynamics and give due consideration to regional factors such as economy, housing, education, medical and environment, thereby ensuring that the policies are well-suited to the region’s actual conditions and needs. Finally, to establish comprehensive childbirth insurance, including medical care and mental health counseling during pregnancy and after childbirth, as well as convenient and accessible childcare services. These measures should ensuring urban and rural families have access to necessary resources. This would be beneficial for achieving regional balance in birth rates and ensuring the healthy development of the population.

Despite the important findings of this study, the limitations should be noted. Firstly, due to the different fertility policies in ethnic minority areas compared to the rest of the country, and the severe data deficiency, ethnic minority areas were not included in this study. This analytical boundary necessitates caution in generalizing findings to contexts with strong ethnic heterogeneity in reproductive behaviors. Secondly, to investigate the impact of the Covid-19 pandemic on birth rates, it would theoretically be necessary to compare data from after the pandemic with data from before the pandemic. However, the data used in this study ends at the end of 2021, when the pandemic was still ongoing. Therefore, it is not possible to accurately estimate the impact of the pandemic on birth rates. Finally, although the fertility policy recommendations are grounded in this study’s findings, the research primarily focused on the spatiotemporal heterogeneity of socioeconomic impacts on birth rates and did not involve empirical evaluation of specific policy interventions. These critical dimensions remain to be examined in our next research.

## Data Availability

The original contributions presented in the study are included in the article/Supplementary material, further inquiries can be directed to the corresponding authors.
