# Supplementary material for: Spatiotemporal heterogeneity of the association between socioeconomic development and birth rate: a geographically and temporally weighted regression modeling study in China
Source: Front Public Health. 2025 May 21;13:1587358. doi: 10.3389/fpubh.2025.1587358 (PMC12133731; doi:10.3389/fpubh.2025.1587358)
Supplement: Supplementary file 2 [file Table_1.docx]

**Table S1 Spatial Nonstationarity Tests of Variables**

| Variable | Interquartile (GTWR) | 2×SE (OLS) | Extra local variation |
| --- | --- | --- | --- |
| LnperGDP | 2.123 | 0.378 | YES |
| Urbanization | 6.222 | 1.360 | YES |
| Incomeratio | 1.073 | 0.280 | YES |
| LnHouse | 1.606 | 0.354 | YES |
| LnAir | 2.382 | 0.320 | YES |
| LnBednum | 1.849 | 0.480 | YES |
| Tecinvestment | 0.317 | 0.086 | YES |
| Eduinvestment | 0.179 | 0.032 | YES |
| LnperBook | 0.546 | 0.224 | YES |

**Table S2 Summary of OLS Regression**

| Variable | Estimate | Std. Error | VIF |
| --- | --- | --- | --- |
| Intercept | 5.172** | 1.867 |  |
| LnperGDP | 1.126 | 0.680 | 3.389 |
| Urbanization | 1.558*** | 0.140 | 3.008 |
| Incomeratio | 0.543** | 0.177 | 1.243 |
| LnHouse | 1.225*** | 0.160 | 2.431 |
| LnAir | -2.993*** | 0.240 | 1.182 |
| LnBednum | 0.221*** | 0.043 | 1.66 |
| Tecinvestment | 0.194*** | 0.016 | 1.744 |
| Eduinvestment | 0.119 | 0.112 | 1.282 |
| LnperBook | -0.319 | 0.189 | 2.387 |
| Adjusted R^2^ | 0.246 | |  |

Note: *** p<0.001; ** p<0.01; * p<0.05; . p<0.1.

**Table S3 Summary of Moran's I**

| Year | Moran's I | Z-value | P-value |
| --- | --- | --- | --- |
| 2012 | 0.393 | 9.805 | 0.000 |
| 2013 | 0.424 | 10.031 | 0.000 |
| 2014 | 0.405 | 9.776 | 0.000 |
| 2015 | 0.511 | 12.329 | 0.000 |
| 2016 | 0.559 | 13.588 | 0.000 |
| 2017 | 0.567 | 14.115 | 0.000 |
| 2018 | 0.640 | 15.838 | 0.000 |
| 2019 | 0.626 | 15.543 | 0.000 |
| 2020 | 0.553 | 13.331 | 0.000 |
| 2021 | 0.643 | 12.974 | 0.000 |
